# Socioeconomic benefits and limited parent–offspring disagreement in arranged marriages in Nepal

**DOI:** 10.1017/ehs.2023.3

**Published:** 2023-02-22

**Authors:** Elizabeth Agey, Savannah Crippen, Alyx Wells, Parash Upreti

**Affiliations:** 1 University of California, Santa Barbara, USA; 2 Independent Researcher, USA

**Keywords:** Arranged marriage, mate choice, parent–offspring conflict, social support, Nepal

## Abstract

Mate preferences probably evolved to increase fitness; however, studies using arranged and non-arranged marriage as proxies for limited and free mate choice (respectively) do not find any reproductive differences. We explore why arranged and non-arranged marriages are an imperfect proxy for limited and free-choice matings and what fitness effects different marriage types could produce. Data from focus group discussions with men and women in Nepal show that there are three spouse choice categories with differing levels of parental influence over mate choice, reinforcing that arranged and non-arranged are not dichotomous. Discussions also show that parents and offspring, especially sons, may be more aligned in in-law/mate preferences than expected, demonstrating the need to establish clear domains of parent–offspring disagreement over spouse choice in the community before investigating fitness. Several social and financial benefits that are only available to arranged couples in this community were detected, and these benefits could compensate for any costs of not choosing a spouse independently. These benefits of arranged marriage are more salient for women than for men. These discussions indicate that predictions about the effects of spouse choice on fitness outcomes may differ for men and women and depend on community-specific socioeconomic benefits.

## Graphical abstract

**Figure:**
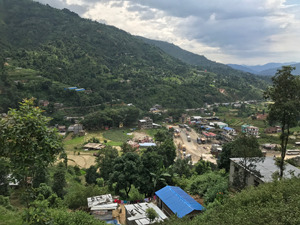

**Social media summary:** Arranged marriages in Nepal have high parent–offspring agreement and come with many socioeconomic advantages.

## I. Introduction

Numerous studies of human mate preferences have identified particular desirable traits and the fitness benefits they supposedly confer on offspring. Yet is there an actual connection between these desirable traits and better fitness outcomes? This underlying assumption, that mate preferences increase fitness, has been tested several times in animal studies, where experimenters either assign a random mate or allow the animal to choose a preferred mate. These experimental studies largely confirm fitness benefits to free mate choice, including offspring viability (Anderson, Kim, & Gowaty, 2007; Drickamer, Gowaty, & Holmes, 2000; Partridge, 1980), growth (Drickamer, Gowaty, & Wagner, 2003; Havens, Orzack, & Etges, 2011) and immune function (Raveh et al., 2014). Parallel attempts to demonstrate similar benefits of free mate choice in humans, using arranged and non-arranged marriages as proxies for limited and free mate choice, respectively (Agey & Gaulin, 2018; Sorokowski et al., 2017), have so far failed to detect differences in offspring survival or in time to first reproduction for couples in arranged vs. non-arranged marriages. There are several fundamental differences between the animal experimental design and human arranged marriages that help explain their discrepant empirical results. In contrast to experimental studies, arranged and non-arranged marriages are not randomly distributed in society. Furthermore, even in cases where offspring have little influence on spouse choice, their parents are still exhibiting mate choice on their behalf.

This article will explore other reasons that comparison of arranged and non-arranged marriages may be an inappropriate proxy for experimental animal studies by examining ethnographic data from focus group discussions in Dhading District, Nepal, a culture with a rich history of arranged marriages. These discussions demonstrate that parents and offspring may have more agreement when choosing an in-law/spouse than previously assumed, that parent–offspring agreement is stronger between parents and sons than parents and daughters, and that arranged marriages can offer benefits that may compensate for any potential fitness loss from not choosing one's own spouse, particularly for women.

Marriages arranged by parents or other kin are widespread (Apostolou, 2007; Broude & Greene, 1983) and have probably existed at least since the first modern humans left Africa (Walker, Hill, Flinn, & Ellsworth, 2011). Arranged marriages vary in the degree of parental involvement: sometimes parents choose mates for their children with no input from the marrying individuals, but sometimes parents get approval on their choice from children before settling a marriage agreement (Shenk, 2017). Arranged marriage rates are higher for women (Broude & Greene, 1983), potentially producing couples where the husband and wife had different degrees of spouse choice; for instance, a husband could independently negotiate his marriage with a bride's parents with little input from the bride. Owing to this asymmetry, the effects of arranged marriage could be more pronounced for women than for men. Arranged marriage is also more common and more parentally controlled in agricultural societies and societies with other forms of inherited wealth (Apostolou, 2010b, 2012), and in these contexts there may be strong economic incentives for arranged marriage.

When arranging a marriage, the interests of parents and offspring are not expected to fully align. Parents should broadly focus on the interests of the family because they can gain fitness equally through each of their children, while offspring gain more fitness through their own reproduction than through the reproduction of their siblings (Alexander, 1974; Trivers, 1974). Parents express stronger preferences for matches that increase or solidify social networks and prestige, as this can increase fitness for all of their offspring (Agey, Morris, Chandy, & Gaulin, 2021; Apostolou, 2008b). Parents also place more emphasis on choosing in-laws from the same ethnic or religious background while offspring place more emphasis on markers of genetic quality, such as physical attractiveness (Agey et al., 2021; Apostolou, 2011, 2015; Bovet, Raiber, Ren, Wang, & Seabright, 2018; Buunk, Park, & Dubbs, 2008; Perilloux, Fleischman, & Buss, 2011). Because some markers of attractiveness are complementary rather than absolute (e.g. Major Histocompatibility Complex discordance which is dependent on one's own genetic profile), parents may be less able to choose the most genetically compatible mates for their children. Parents may also prefer that their children, especially daughters, marry at younger ages than offspring prefer because it allows parents to exert more control over spouse choice (Apostolou, 2010a). Because arranged marriages are more common for women than for men, daughters may thus achieve their own mate preferences less frequently.

While there is scope for disagreement between parents and offspring, there should also be significant overlap in their interests. Surveys have found agreement between parents and offspring over traits like kindness, personality and intelligence (Apostolou, 2011; Fugère, Doucette, Chabot, & Cousins, 2017; Perilloux et al., 2011), and greater general agreement in collectivist cultures (Guo, Li, & Yu, 2017). Additionally, parents could have greater ecological knowledge or experience that makes them well suited to find quality partners for their offspring. The more regular pattern in cultures with arranged marriage may be for parents to find a spouse for their offspring that meets all parties’ criteria. Parents and offspring may also be willing to compromise on less important traits provided that each of their strongest preferences are met. If parents and offspring often cooperate or compromise, then their disagreements may have limited fitness effects.

In many cultural contexts, the social, financial or resource-related benefits predicated on an arranged marriage may further push offspring to cooperate or compromise with their parents over spouse choice. Because parents are older and often control access to property or social networks, individuals who marry against their parents’ wishes may face strong sanctions from parents and/or the community. Parents may threaten to withhold the family's resources (e.g. property or money) if their children do not agree to an arranged marriage (Agey et al., 2021). Offspring that have arranged marriages also have greater access to alliance, trade and kin networks than do their peers in non-arranged marriages (Wiessner, 2009). Ostracism from parents, kin and community may also reduce the scope of alloparental care, especially from grandparents. Because grandparental care has been tied to offspring survival (Sear & Mace, 2008), parents may be able to encourage arranged marriages by threatening to withhold grandparental care from children of non-arranged marriages. Since maternal grandparents seem to have the greatest impact on survival, women may be further incentivised to accept arranged marriages in cases where their parents may withdraw support. Thus, while preventing individuals from choosing a mate independently could reduce some genetic fitness components, parental choice via arranged marriage may provide many other benefits that could compensatorily enhance fitness.

In order to fully comprehend the ways in which spouse choice may affect fitness, it is important to understand (a) the ways in which spouses are chosen, (b) the qualities that may produce disagreement and (c) the costs and benefits of different modes of spouse choice in the community of interest. Using in-depth focus group discussions from a community in Dhading District, Nepal, we will explore these three topics and how they might affect fitness. These data will illuminate the reasons why comparison of arranged and non-arranged marriages does not show the same fitness differences as limited and free mate choice in animal experiments. It will also demonstrate the types of ethnographic work that must be done in order to make meaningful predictions about the fitness consequences of spouse choice in a particular community.

## II. Methods

### A. Study population

Many populations in Nepal traditionally practise arranged marriage. However, over the past 50 years Nepal has begun a dramatic shift in marriage practices, including fewer child and adolescent marriages, more inter-caste marriages and greater offspring participation in spouse choice (Ghimire, Axinn, Yabiku, & Thornton, 2006). This shift has produced a cohort of individuals in Nepal whose marriages vary from arranged to non-arranged. In addition to this significant variation in marriage type, the national divorce rate is less than 1% and remarriage, especially for women, is uncommon (*Nepal Population and Housing Census 2011*, 2012). Thus, choosing a spouse is effectively permanent, making this a useful context in which to examine the effects of different modes of spouse choice.

In many castes and ethnic groups in Nepal, marriages are traditionally restricted to members of the same caste and subcaste, but not within the same *gotra* (lineage). Caste is primarily inherited through males, but there are also subcastes that are specific to intermarried couples that can change the caste of their children. For example, the Khatri Chhetri subcaste denotes the presence of an intermarriage between Brahmin man and non-Brahmin woman (Höfer, 1979). Parents may informally recruit a *lami* (matchmaker), usually extended kin or acquaintances who live in other locations, to help search for suitable spouses. After marriage, women traditionally live with their husband and his family. Historically, dowry was an important part of marriage negotiations, providing financial transfer from the bride's family to the groom and his family. While the practice has been illegal in Nepal since 2009, the law primarily prevents the groom's family from requesting specific items rather than preventing significant marital gift giving. While daughters traditionally receive financial transfers at marriage, sons primarily receive financial transfers through inheritances. Prior to 2017, Nepali law required inheritances to be split equally among all sons but did not require any transfer to daughters (National Civil Code Act, 2017). Nepal does not legally allow marriage until age 20 for either men or women (National Civil Code Act, 2017). Most marriages adhere to this law, but it is not always well-enforced, especially in rural areas where marriages are not always registered with a government office.

**Figure 1.**
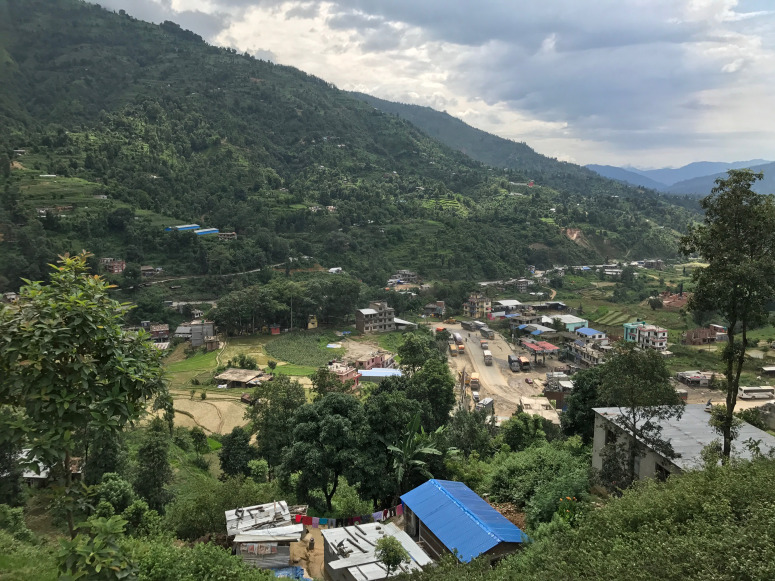
Image of the study site in Dhading, Nepal, showing the highway that passes through the town, terraced fields and hilly terrain.

The data for this study were collected in a large community (over 20,000 people) in the Dhading District of Nepal, which lies directly west of the Kathmandu Valley. The community has access to a major highway, and socioeconomic status varies with proximity to the highway. Most individuals in the community are farmers or shopkeepers, and women traditionally work within the household. Many people under 30 years of age have achieved the equivalent of a high-school education or beyond, but education levels decrease with age and for women. The community includes members of a variety of castes and ethnic groups, with the highest representation coming from Brahmins, Chhetris, Tamangs and Newars. While most marriages in the community adhere to the national law about marriage age, some people in the community, particularly women, marry as teenagers. Marriage under age 16 is rare among individuals married in the last 40 years. Based on data we collected in the community, 78.6% of people in the oldest generation have said their spouse was primarily chosen by their parents. Of people married in the last 15 years, only 56.2% said their parents were primarily responsible for their spouse choice, mirroring the widespread shift in Nepal from arranged to non-arranged marriages. The variation in marriage type within a single generation makes this community a good place to examine the diversity of methods of spouse choice and explore their potential fitness effects (Figure 1).

### B. Data collection

Semi-structured focus group discussions were conducted in 2019 and 2022. Participants were recruited via convenience sampling in various neighbourhoods within the community, and recruitment was done at different times of day to target people with varying work schedules. Participants were told they could bring a friend or neighbour of the same gender and approximate age to participate in the group as well, in the hopes that some familiarity within the group would facilitate more casual and lively conversations. A few (three or four) women across these groups brought mothers- or daughters-in-law to participate; in these cases, they were placed into separate groups based on age. All participants were recruited by local research assistants, as previous experience showed increased trust and willingness to participate when local assistants were involved. Men and women were recruited from three life stages that roughly mirror age cohorts: never-married adults ages 18–30, adults married within the last 10 years and married adults with adult children (hereafter referred to as unmarried, recently married and older, respectively).

Individual focus groups comprised homogeneous gender and age groups (e.g. older women). Discussions were facilitated in Nepali by a trained local research assistant of the same gender as the participants and were limited to 1 hour. In total, we conducted 19 focus groups with an average size of 5.6 participants. The number and composition of focus groups can be found in Table 1. Most of the unmarried individuals were students at the time of the discussions (50%). A majority of the married men ran local businesses (52%) or were in the service industry (33%). Most women interviewed were either farmers (60%) or shopkeepers (33%). While participant incomes were not collected, generally those who work in business or run shops tend to have higher incomes than farmers or those who do service jobs.

**Table 1.** Focus group summary statistics for each demographic category

Groups	Number of groups	Total respondents	Mean age	Mean number of children	Mean age of children
	*Men*				
Unmarried	2	12	21.9	0	NA
Recently married	2	13	34.2	1.5	5.5
Older	2	11	49.0	2.75	19.9
	*Women*				
Unmarried	4	25	19.56	0	NA
Recently married	4	26	33.4	2.0	13.1
Older	3	19	47.8	2.9	26.4

Note: ‘Unmarried’ includes adults aged 18–30 who have never married, ‘Recently married’ includes individuals of any age married within the last 10 years, and ‘Older’ includes ever-married adults with children aged 16 or older. All ages are in years.

Focus groups were designed to target topics that were relevant to each group's life stage. The facilitators were given a list of potential questions and vignettes to generate discussion but were instructed to allow conversation to flow naturally and alter the questions or their order accordingly. Unmarried adults were asked questions about dating, the marriage process, desirable qualities of potential spouses and relationships with their parents. Recently married adults were asked about the process of finding a spouse, their lives after marriage, and about starting a family. Older adults were asked questions relating to their children's marriages (or future marriages) and their own lives after marriage. Many discussion topics were chosen to target our specific research questions:

A: What types of marriages exist in the community and what are the characteristics of each (i.e. is spouse choice dichotomous)?

B: Do parents and offspring agree over the ideal qualities of an in-law/spouse, respectively?

C: What kinds of social and financial outcomes are tied to spouse choice decisions?

Most of the questions prompted participants to think about the community generally rather than about their own experiences to increase openness, especially surrounding more difficult or taboo topics, such as dowry, marital disagreements, affairs and fertility.

All focus groups were recorded, transcribed in Nepali, and translated into English by a bilingual native Nepali speaker. The Nepali–English translations were checked by a second bilingual native Nepali speaker (Author P.U.) to ensure their accuracy. Translations were also checked to make sure that important context was not lost, paying close attention to culturally specific words or phrases that have implied meanings or do not translate literally. Where necessary for clarity, notes on such meanings are inserted into quotes in square brackets.

### C. Data analysis

English transcripts were read and independently coded for content by S.C. and A.W. When a new topic was identified, it was added to a codebook and given a brief description to maintain consistency of use in future coding. The coders then cross-checked each other's work for accuracy and comprehensiveness. All authors read the transcripts in full, and each author independently kept notes on themes they noticed, statements that were surprising and clear differences between demographic groups.

After this initial coding, statements were reorganised by code with tags denoting the gender/age group where it originated. Following the reorganisation, all statements from each theme were read together. The authors noted any differences based on age or gender, as well as other distinctions drawn by participants, including statements about different behaviours among the rich/poor, rural/urban or past/present. These themes and patterns were discussed in biweekly meetings in which the authors compared their observations with each other's observations, as well as to their own notes from earlier readings of the transcripts. The patterns and themes described in this analysis were agreed upon by all authors.

## III. Results

### A. Is the mode of spouse choice a dichotomous variable?

Focus group discussions identified three types of spouse choice in marriage: arranged marriages, love marriages and elopements. Each vary on the autonomy of the spouse-choice decision (summarised in Table 2).

**Table 2.** Summary of marriage types and their associated forms of support

	Arranged marriage	Love marriage	Elopement
Who initially finds spouse?	Parent(s) or other relatives	Offspring	Offspring
Does the other party approve of spouse choice?	Offspring may or may not approve	Yes	No (or parents were never asked)
Financial support	Dowry/gift giving, inheritance	Dowry/gift giving, inheritance	Inheritance
Social support	Strong, family can mediate conflicts, better relationships with in-laws	Medium, acceptance from family and community but less family involvement	Low for months to years after marriage
Grandparental support	Strong desire to invest in grandchildren, pressure to quickly have children, more likely to reside with grandchildren	Desire to invest, but less pressure to quickly have children, may or may not reside with grandchildren	Elopements forgiven after grandchildren are born, but investment may be limited by separate residence from grandchildren

#### i. Arranged marriages

Arranged marriages were characterised as marriages in which parents (or other relatives) find and select a potential spouse for their children. This may or may not include a child's approval of the parent's choice. While respondents also sometimes used the term ‘arranged marriage’ to refer to cases in which a family approved of a spouse the child chose for themselves, such cases more closely resemble the description of love marriage, discussed in more detail below. Arranged marriages were categorised as a form of respect and obedience towards parents by some respondents, especially unmarried women (Supplementary Information, quote A1). Arranged marriage was seen by almost all groups as a contract between the whole families of the bride and groom. Families are deeply involved in choosing and approving a spouse, the marriage ceremony and post-marital life, for example:

If you find a husband yourself, then whatever happens between the two of you cannot be shared with other people or your family because you are the one who chose him. But if it is arranged and you don't like your husband, and if he does not treat you well, you can yell at your parents, ‘what kind of man did you get me?’ (Audience laughs; recently married woman)

Respondents identified types of people who were more likely to engage in arranged marriages. The groups noted that children who were passing the optimal marriage age were more likely to have arranged marriages. Participants identified two primary reasons why an individual may not be married by this time: individuals may be too timid to find a partner independently or individuals may have dated a self-chosen partner but experienced a difficult breakup.

Arranged marriages were repeatedly identified by respondents as the most common type of marriage in the community and across socioeconomic groups, but many of them noted that love marriages are becoming more common.

#### ii. Love marriage

Love marriages were described by respondents as cases in which children found someone they would like to marry and sought parental approval for the marriage. Following parental approval, these matches are formalised through the same traditional marriages rites and ceremonies that occur in arranged marriages. Thus, love marriages and arranged marriages differ primarily by who initially finds the spouse. Parents seem to be relatively accepting of their child's wishes in these types of marriages (Supplementary Information, quotes A2 and A3).

Love marriages are characterised as more independent than arranged marriages, and this independence spanned the entire process from choosing a spouse to post-marital life. Even if parents agree to the child's spouse choice, they do not see themselves as having rights to intervene in the couple's life. Most respondents said that love marriages are becoming increasingly common, with a few respondents saying this is now the most common type of marriage occurring in the community.

#### iii. Elopement

The last type of marriage identified by respondents was elopement. This type of marriage was described as running away to get married without any parental approval. This could occur after a rejection or an anticipated rejection of a potential spouse by parents. These marriages were often described as a form of disobedience or extreme independence. Elopements are very often tied to breaking societal marriage rules, including marrying outside of the proper caste.

Some respondents noted that elopements are a good way to save money that would typically be spent on a wedding, indicating that elopements may be more attractive to people who cannot afford a wedding ceremony. However, the overwhelming majority of people characterised elopements as being very undesirable, among both family members and the community. Some respondents stated that love marriages may sometimes be accepted just to avoid the stigma of an elopement:

Parents are also giving them permission to do love marriages to secure their reputation. They're giving their children permission so they don't elope or their parents don't have to feel insulted in society. (Unmarried woman)

Despite this undesirability, individuals also reported that elopements are very often accepted after some time, especially after the couple has children (Supplementary Information, quote A4). Elopements were continually described as rare events, and the least common type of marriage.

### B. Do parents and offspring agree on the ideal qualities of an in-law or spouse?

**Figure 2.**
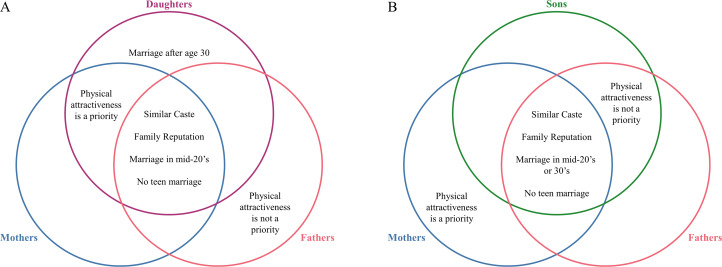
Potential for parent–offspring agreement and disagreement over traits in a potential spouse. These diagrams represent the potential for disagreement between daughters and their parents (a) and sons and their parents (b) based on focus group discussions with unmarried and older participants. Areas of overlap represent agreement on the listed traits, while non-overlapping areas represent disagreement. (a) Daughters, mothers and fathers agree that a potential spouse should be of a similar caste and come from a family with a good reputation. They also agree that marriage should occur in the daughter's mid-20s and not occur in their teenage years. Mothers and daughters agree that physical attractiveness is desirable and important in a potential spouse. Daughters sometimes express a preference to marry after age 30, which would not be desirable for mothers or fathers. (b) Sons, mothers and fathers agree that a potential spouse should be of a similar caste and come from a family with a good reputation. They should also agree that marriage should occur in the son's mid-20s or 30s and not occur in their teenage years. Sons and fathers agree that physical attractiveness should not be a priority for choosing a spouse, while mothers think that physical attractiveness is desirable and important.

The focus groups were asked to describe what qualities make a good spouse, or what qualities they would look for in a spouse for a son or daughter. Respondents freely listed 26 different qualities. A brief description of the data across all desirable qualities is included in Supplementary Table S1, but only traits that were expected to produce disagreement based on previous research on parent–offspring disagreement over spouse choice (Agey et al., 2021; Apostolou, 2010a; Buunk et al., 2008; Perilloux et al., 2011) are discussed below. The results are summarised in Figure 2.

#### i. Caste

Caste, which is associated with the same religion and cultural/ethnic group in this community, was an important factor in spouse choice for most groups, and many people identified it as the very first trait they would consider in a potential spouse. Participants commented that a bride and groom's castes should match, and that lower-caste brides would face discrimination by their in-laws after marriage. Marrying someone of a different caste was also deemed unacceptable to society generally, so the stigma seems to extend beyond the family unit.

Older participants pointed out that younger individuals or individuals choosing their own spouses are less likely to care about caste (Supplementary Information, quote B1). However, several unmarried respondents identified caste as an important factor in spouse choice. They also recognised the importance of caste to their parents and showed willingness to discuss and compromise with their parents if they wanted to marry someone of a different caste (Supplementary Information, quote B2). Some parents showed a similar willingness to compromise on the issue of caste, for example:

Well, if they are from lower caste, then the community might not respect us. But if they can't live without each other, then we have to go with them. (Older woman)

Some older individuals said inter-caste marriages (often elopements) would eventually be accepted if the couple really loved each other and/or if they have children. Overall, these discussions indicate more alignment of interests between parents and offspring, as well as more willingness to compromise, than anticipated based on previous research.

#### ii. Physical attractiveness and health

Attractiveness was mentioned by many groups, but women seemed to mention physical attractiveness as a desirable trait more than men. Women across all age categories listed attractiveness as a desirable quality in a husband, daughter-in-law and/or son-in-law, although it was often not the first quality listed. Men, on the other hand, repeatedly stated that physical beauty should *not* be a priority in marriage, if they mentioned it at all. Several unmarried men pointed out that beauty fades over time, and that other qualities are much more important (Supplementary Information, quotes B3 and B4). Men stated that physical attractiveness should not be a priority even in non-arranged marriages:

If you fall in love with someone then you marry her. If you really love the woman not for sex but for her characteristics, her emotions, the way she presents herself then your marriage will be successful. But if you only marry a woman for sex then in the long run it might not be successful. (Recently married man)

Most older men mirrored this sentiment, saying that beauty was not enough to make a good daughter-in-law and that a son-in-law did not need to be handsome as long as they can take care of their daughter (Supplementary Information, quote B5).

While physical attractiveness may not be a priority for men, one group of unmarried men did mention that a potential bride should be free of disease and long-term health issues. This, combined with discussion of finding an ‘average looking’ girl, may indicate that these men have a minimum threshold of physical attractiveness. Women of all ages in the community appear more concerned about physical attractiveness than men, possibly indicating some disagreement between daughters and their fathers.

#### iii. Family status, reputation and affluence

The quality of the potential bride's or groom's family was commonly identified as an important factor in spouse choice. A ‘quality family’ has wealth and property, exhibits good behaviours (e.g. they do not fight with themselves or others, they refrain from drinking), and is well regarded by others. ‘Good behaviour’ and ‘high status’ were identified multiple times as more important than the family's wealth, although familial wealth and property were still important. Wealth was repeatedly identified as a bargaining chip used by parents and matchmakers to convince a man or woman to accept an arranged match.

Unmarried men and women listed family reputation as a desirable quality when considering a wife or husband; however, when unmarried women expressed a desire for grooms from good families, they also clarified that the groom himself should have good behaviours. Women repeatedly stated that family status was not always a worthwhile quality to consider because it cannot make up for bad qualities of a groom.

Sometimes the person chosen by parents might have vices, like drinking, smoking, or violence, and choosing by yourself is a better option. (Unmarried woman)

This indicates that women, while they do value good family background, only perceive family background as a reliable signal of quality when the groom is also well behaved.

Older men and women also placed high value on the quality of the family of a potential bride or groom. They characterised the quality of the family as a trait to check prior to any discussion of marriage (Supplementary Information, quote B6). The focus on family quality by parents may cause some disagreement with offspring, particularly daughters, who may weigh the behaviour of the potential groom more than the quality of his family.

#### iv. Age at marriage

In the focus groups, almost all ages and genders stated that the ideal age of marriage is between 20 and 30 years old, but most responses were concentrated in the 20–25 age range. Many respondents, including both men and women, stated that finishing school and getting a job is important to do before considering marriage. Virtually all participants agreed that marrying as a teenager or before maturity is not ideal. Marrying as a teen was repeatedly tied to elopements in these discussions. Many older individuals pointed out that age and maturity were major factors in whether love marriages would be successful, indicating that parents may be less likely to accept non-arranged matches if they perceive their offspring to be too young (Supplementary Information, quote B7). However, arranged marriages in the community sometimes also take place at ages younger than the stated ideal marriage age. Some of the women expressed discontent about having arranged marriages at young ages (Supplementary Information, quote B8). Because marriage ages have increased in Nepal, owing to both secular trends and the passing of laws about marriage age, it is possible that disagreements over marriage age in arranged marriages are decreasing.

While there is general concern over marriages taking place too young, older individuals also expressed concern about waiting too long for marriage, particularly for women. Conversely, several unmarried women emphasised their desire to have a career, become independent and marry in their mid or late twenties. Unmarried women noted the disconnect between parental desires and their own desires:

When we turn 20 or 21 our parents start to talk about marriage and they'll start to look for a groom. But we don't want to get married earlier, so we'll get married after we are 30 years old. (Unmarried woman)

There is also a gender difference in preferred marriage age in this community, as some respondents stated that a 3–4 year age gap between husband and wife is ideal (but note that women did not prefer *too* large an age gap). Two groups also pointed out that men are mostly immune from family pressure to marry, while women are more likely to face pressure. Thus, there may be disagreement between women and their parents when it comes to marriage timing. For example, if a man wants to marry at 23 years old, then his potential wives may be only 19 or 20, which is below or at the bottom the women's stated ideal age range for marriage. If women simultaneously face more pressure to marry at younger ages than men do, the conflict between their stated ideals and parental pressure may lead to conflict.

### C. What kinds of social and financial effects are tied to spouse choice decisions?

It is possible that allowing parents to choose a spouse, or at least seeking their approval for a spouse, is tied to certain benefits for the couple that would be lost if parents are not involved in the spouse choice. The results below are summarised in Table 2.

#### i. Family and community support for the marriage

Elopements, especially inter-caste elopements, were consistently identified as undesirable or unacceptable, and they elicit less family and community support than arranged or love marriages. Respondents repeatedly stated that these consequences seem to be directed towards women who elope more than men who elope.

Towards the daughter-in-law who had an elopement marriage, parents might love and respect her less but towards a son there is no discrimination. (Unmarried man)

Several circumstances lead to the acceptance of eloped couples, including having children, becoming financially independent or a bride fulfilling the expectations of her in-laws. But even with parental acceptance, many groups stated that couples who eloped cannot complain about their marriage since it was their choice. Marriages that were based on love may also still face increased conflict, especially between mothers- and daughters-in-law:

I think in marriages that were not arranged, daughters-in-law and mothers-in-law fight more. In an arranged marriage, the daughter-in-law knows that the family is chosen by her parents, and if she argues, then it does not show good upbringing. But in love marriages, it is the son who brought her in the house. So, the son is more inclined to his wife and therefore the wife has power and support to quarrel with her in-laws. (Recently married woman)

The timeline for which elopements are accepted varied widely between respondents, ranging from a month to 10 years, indicating varying degrees of social sanctions for eloping.

#### ii. Financial benefits

As mentioned briefly above, there is a general expectation in this community that eloping couples should be more financially independent than their arranged-marriage counterparts. While some couples may elope to avoid large wedding costs, there may also be financial benefits tied to arranged marriages (described below) that eloping couples are less likely to receive.

While dowry has been illegal in Nepal since 2009, the practice still exists in various forms and areas of Nepal. Many respondents identified that traditional dowry (gifts from the bride's family that were requested by the groom and his family) has been replaced by gift-giving at the discretion of the bride's parents. There are a variety of gifts given to the bride at the time of marriage, even if these are not considered a formal dowry. Some commonly identified items were TVs, furniture, clothing, appliances, gold jewelry and automobiles. Gifts may also include cash or loans to a groom to start a business. These gifts belong exclusively to the bride, and by extension the groom, except in the case of large, shared items (i.e. a refrigerator would be shared by the groom's family and thus would have more ambiguous ownership). Some participants noted that dowry items used to be owned by the groom and his parents, but this view has mostly disappeared. One respondent stated that, in the case of divorce, a bride could take back all her dowry/gifts.

Gift-giving and/or dowry was consistently identified as more prevalent in arranged marriages, as it is part of the formal marriage negotiations and ceremony. Women in these focus groups tended to perceive dowry as staying the same, increasing or being informally required despite its illegality (Supplementary Information, quotes C1 and C2). While men identified a shift from dowry to gift-giving, they consistently flagged dowry as decreasing or absent in the community (Supplementary Information, quote C3). Most instances where women identified dowry as decreasing or absent were in discussing their own marriages or opinions, not when discussing the community generally (Supplementary Information, quotes C4 and C5).

In Nepal, inheritance is another common way through which financial resources and/or property are transferred. Inheritances are traditionally given to sons, although daughters have historically sometimes been given property, especially if a couple has no sons. While current Nepali laws mandate that all sons and daughters in a family are entitled to equal inheritance (National Civil Code Act, 2017), only sons were legally entitled to inheritances before 2017, the time during which most of the married individuals in these discussions would have been married. Based on these data, unmarried men are aware of the laws regarding inheritance (Supplementary Information, quote C6), and thus it may not be a decision factor when choosing a spouse. Inheritance was not discussed by any women in these groups.

#### iii. Grandparental Investment

Women and older men noted that couples in arranged marriages face significantly more pressure from parents and in-laws to have children quickly after marriage than do couples in other types of marriages. However, recently married men explicitly stated that they do not face pressure to have kids earlier, suggesting a gender difference in parental pressure.

In arranged marriages, couples have kids earlier than in love marriages because there is pressure from parents to have kids as soon as they get married because they want to play with their grandkids before they die. But in love marriage, parents think that the couple has decided their life planning themselves and will let them do however they want to live their life. (Older man)

Since arranged marriages were routinely characterised as having more family involvement and a higher sense of familial obligation than love marriages and elopements, this increased parental pressure to have children is not surprising. Furthermore, this pressure seems to stem from a strong desire to invest in grandchildren as soon as possible. While respondents repeatedly said that parents accept elopements following the birth of the couple's first child, there does not appear to be the same pressure to have children quickly in those marriages.

Direct grandparental investment may also be dependent on proximity. In Nepal it is common for sons to co-reside with their parents and care for them in old age. Participants indicated that it is the parents’ choice of which of their sons (and, by extension, daughters-in-law) they live with, often preferring to live with the youngest son. However, respondents indicated that wives could similarly control whether or not they continue to live with their husband's parents:

Sometimes we have to split with our parents and live with our wife and children. It normally happens because the wife does not like to live with our parents after marriage because she might feel that she is not getting more privacy, or she just wants to live with her husband and children and live life accordingly. Wife is a catalyst. (Recently married man)

Older participants repeatedly stated a strong desire for daughters-in-law that would respect them and take care of the household (see Supplementary Table S1), indicating that this is a salient factor when arranging a marriage. Furthermore, as pointed out in multiple groups, mothers- and daughters-in-law are more likely to quarrel when there was an elopement or love marriage. It is therefore plausible that parents prefer to live with sons who had arranged marriages. This would lead to additional direct grandparental care being targeted to grandchildren from arranged couples. The relationship between marriage type and indirect grandparental investment, however, cannot be resolved from these discussions alone.

## IV. Discussion

### A. Spouse choice is not a dichotomous variable

It is clear from these discussions that arranged and non-arranged marriage do not represent a dichotomy. Participants identified three types of marriages but described variation within each type. Arranged marriage may or may not have offspring approval of the parents’ choice, implying that a broad category for ‘arranged marriage’ may obscure important variation in the degree of choice offspring have. Both love marriage and elopement would technically be considered non-arranged marriages, but love marriage and elopement have very different levels of parental involvement before and after marriage. Thus, sorting marriages based on who initially found the spouse may still not capture the full range of variation in spouse choice decisions. In Dhading, spouse choice may be best measured as an index of several variables, including who originally found the spouse, who initially suggested marriage to that person, whether permission was sought and granted from other parties prior to marriage, and the perceived degree to which each party was able to influence the other in their choice. Furthermore, information offered by participants suggests that husbands and wives may answer questions about spouse choice differently. Approval for a marriage could come from the bride's family, the groom's family, or from both families, and each of these situations could have different implications for the couple's post-marital life. Failing to capture these sources of variation may make it difficult to accurately differentiate the consequences of partner choice.

### B. Parents and offspring often agree over the ideal qualities of a spouse

Older respondents were more likely than unmarried respondents to name caste as the first quality they would consider in a spouse for their children. While this may indicate that parents care more about caste than unmarried individuals, there was more willingness to compromise on caste-matching than expected. Parents may face a difficult choice when their children want to marry someone of a different caste because, if parents do not approve of an inter-caste match, the couple may elope instead. Parents may thus be willing to approve an inter-caste love marriage if it produces less stigma than an inter-caste elopement.

While not discussed by participants in these focus groups, the level of compromise over caste probably varies with the size of the gap between the two castes or ethnicities (which are both integrated into a singular system in Nepal). In Nepal, the phrase *pani chalne* indicates castes and ethnicities designated as ‘pure’ enough to drink water given by them. The lower disadvantaged (i.e. Dalit) castes are excluded from this category, but other ethnic groups are considered *pani chalne* (Höfer, 1979). In informal conversation in this community, we have heard parents say they would accept any marriage from a *pani chalne* group. Therefore, it is likely that a marriage between the most advantaged caste levels would be more acceptable than an inter-ethnic marriage between two *pani chalne* groups, and both of those matches would be more acceptable than a marriage where only one party is in a disadvantaged caste. Because caste is passed primarily through males and hypergamy is formally permitted (Höfer, 1979), parents may be more accepting of sons marrying a lower-caste woman than a daughter marrying a lower-caste man. Thus, women probably face greater sanctions than men for marrying below their caste.

Despite physical attractiveness producing disagreement between parents and offspring in other studies (Agey et al., 2021; Apostolou, 2008a; Bovet et al., 2018; Buunk et al., 2008; Perilloux et al., 2011), these discussions do not indicate strong conflict over this quality. For unmarried men, attractiveness seemed to be a threshold variable: as long as a minimum standard was met, they could be satisfied. The desire for a beautiful daughter-in-law that many older women voiced may mean that sons are able to easily achieve this threshold, even in arranged marriages. Younger women also expressed a desire for physically attractive husbands, indicating that women and their mothers agree in this community. However, older men explicitly stated that a potential husband does not need to be handsome as long as he can provide; thus, disagreement over physical attractiveness is more likely between fathers and daughters than between daughters and mothers or sons and either parent in this community.

Older individuals appeared slightly more attentive than unmarried individuals to the quality of the potential spouse's family. However, unmarried people, especially women, were very clear that the behaviour of the intended spouse, especially around drinking or affairs, was *more* important than the quality of the family; for the marrying individuals a bad apple from a good tree was not an acceptable partner. Parents are probably more attentive to the social network and prestige benefits of arranging matches for their children, and they may sometimes perceive this as being more important than the quality of the potential spouse themselves. Parents might also be worse than offspring at obtaining truthful information about the behaviour of a potential spouse (e.g. because such behaviours may be hidden from older individuals in the community). Where there is discordance between the quality of the family and the quality of the potential spouse, there will probably be more disagreement between parents and offspring. However, in cases where the potential spouse's behaviour is acceptable, then an arranged marriage would satisfy the preferences of both parents and offspring.

There may also be disagreement over marriage timing, especially between parents and daughters. Parents could be pushing daughters to marry earlier than sons for several reasons – female reproduction is more closely tied to age, dowry is also traditionally lower for younger brides, and parents can also exert more control over spouse choice when offspring are younger. While older individuals expressed a lot of concern about very young people eloping, they did not seem as concerned about marriage age in arranged marriages. This indicates that parents may be hesitant to allow love marriages to proceed if they consider their children to be too young or immature to choose their own partner. Parents may reject love matches when their offspring are under the parents’ ideal marriage age. Conversely, young women facing strong parental pressure to have an arranged marriage before they are ready may have to choose between agreeing to their parents’ choice or eloping with a man of their choosing. These factors may explain why elopement is often done by young people. This pattern mirrors patterns of early marriage in Tanzania, in which parents sometimes arrange early marriages to secure socioeconomic stability for their daughters while at the same time being concerned about non-arranged early marriages that appear unstable or based on poor decision making (Baraka, Lawson, Schaffnit, Wamoyi, & Urassa, 2022). Many young women in Dhading expressed a desire to finish school, build a career, and become more financially independent before marriage. They may see these activities as a way to increase their own status, which could be converted to better matches (Schaffnit et al., 2022), more extensive kin networks (Shenk, Towner, Voss, & Alam, 2016), more parental investment and better outcomes for their children (Alami et al., 2020). Because, owing to legal changes and societal trends, marriage age has been steadily increasing in this community (an offspring preference), this could indicate that more compromise between parents and their children over marriage age may be occurring in recent years.

The overall picture shows some avenues for parent–offspring disagreement, but also shows many opportunities for cooperation and compromise. Parents and their children may work together to meet minimum standards on physical attractiveness, quality of behaviour and family background. Parents may compromise on inter-caste marriages in cases where castes are similar or where elopement is a risk. While disagreement about marriage age may also be common, the levels may be decreasing as average marriage age rises in Nepal. Thus, disagreement may only be present in some parent–offspring dyads, and the risk of disagreement over particular qualities is probably asymmetrical for sons and daughters. Likewise, individuals who are shy or do not have their own prospects may obtain better mates through arranged marriage than if they choose on their own, producing very little disagreement. Similarly, individuals who can independently obtain high-quality mates may be able to meet both theirs and their parents’ priorities, again producing very little disagreement. Because elopements (which indicate disagreement) were consistently reported as rare in discussions, it could be the case that parents and offspring are often able to agree on spouse choice in this community. Thus, in cases where both parties cooperate to choose a spouse (i.e. when parents choose a spouse and the offspring approve or when offspring choose a spouse and parents approve), there may be few fitness consequences.

### C. Arranged marriages receive compensatory benefits

Generally, those who choose their spouse with no parental input seem to have less access to family support, at least immediately following their marriages. The consequences of eloping appear stronger for women than for men, as many respondents directly stated that daughters-in-law face more discrimination for eloping than sons-in-law. Even if parents later accept an elopement, it is clear that there is less support if the marriage is unsuccessful or faces hardships, as many participants noted that family cannot get involved in conflicts between self-selected spouses. This decreased familial involvement may have significant ramifications in instances of abuse and intimate partner violence, particularly for women, whose statements in these discussions indicated their desire for strong family support. Dowry, which is unlikely to be paid in elopements, was associated with better relationships with in-laws after marriage, consistent with other studies examining relationships between daughters- and mothers-in-law in Nepal (Diamond-Smith, Dahal, Puri, & Weiser, 2020). This could be because marital transfers can help legitimise new couples in the eyes of the family and community, as bridewealth appears to do in Ghana (Akurugu, Dery, & Domanban, 2022). The lack of dowry could therefore produce more conflict, additional stress, or poorer access to resources in the marital household if the couple lives with the husband's family. Respondents noted that husbands were more likely to take their wife's side during family disagreements in eloped and love marriage couples. While this could attenuate some of the stress and improve treatment of a new wife in her husband's family home, this situation was also tied to the couple establishing a separate nuclear household, thereby further distancing themselves from family. From these lines of evidence, women who choose their own spouses may suffer in terms of support from both her own family and her husband's family. This reduction in material support or increase in interpersonal conflict could increase undernutrition and psychosocial stress, affecting health, pregnancy and birth outcomes (Hobel, Goldstein, & Barrett, 2008; Schneiderman, Ironson, & Siegel, 2005). Since families are more likely to accept an eloping couple following the birth of a child, the effect of reduced familial support may be the strongest on first births.

Arranged couples, who regularly receive gifts/dowries, may be able to achieve financial stability more often or more quickly than couples that eloped, who are unlikely to receive dowries or gifts at marriage. Marital gifts contingent on approval from the bride's family can contribute to the financial stability of the new couple, especially when they consist of cash, land or vehicles that can increase access to jobs and markets. The relationship between marriage type and financial support is less clear for men in this community. While men have always been legally guaranteed to receive an inheritance from their parents, parents may distribute inheritances at different times. If the timing of inheritance is tied to marriage type, that opens an avenue through which resources could be affected by marriage type for men in this community.

Eloping couples were expected to be more financially self-sufficient, and their parents’ acceptance of the marriage was often tied to the couple's financial independence. If eloping couples have less assistance financially from the bride's family (e.g. less gift-giving/dowry) or a delay in assistance from the groom's family (e.g. delayed inheritance), they may take longer to achieve a similar socioeconomic status to arranged couples. Reduced financial resources could lead couples to delay reproduction, resulting in longer first birth intervals for eloped couples. Because women and men face asymmetrical financial sanctions for eloping, it is possible that the effect of elopement on reproduction could differ based on whether the brides’, groom's or both families oppose the marriage.

Further investigation of the types and amounts of grandparental investment by marriage type is required to make predictions about fitness effects. Elopements are often accepted following the birth of a grandchild, and this is due to a desire to invest on the part of the grandparents. However, if couples who elope are more likely to live separately from their parents owing to increased tension between daughters- and mothers-in-law or owing to general expectations that eloping couples should be independent, then they may receive less direct grandparental investment simply as a result of distance (rather than the grandparents’ refusal to offer care). Eloped couples may also receive less grandparental investment if they have children later than couples in arranged marriages because the grandparents are older and less vital or even deceased. Perhaps grandparents who cannot invest as much time in directly caring for grandchildren invest in indirect ways, such as financial support for education expenses. Thus, to test whether there are differences in grandparental investment based on spouse choice, dimensions of both direct and indirect investment need to be measured.

### D. General discussion

Overall, these discussions indicate a scope for gendered conflict over spouse choice decisions. Cross-culturally, studies demonstrate that women are more likely to have their marriages arranged than men (Broude & Greene, 1983). In this community women appear to have more disagreement with their parents over the qualities of a potential spouse, but at the same time have more socioeconomic benefits to gain from arranged marriage. On the other hand, men in this community appear to have high levels of agreement with their parents on spouse qualities and receive financial and social support regardless of marriage type. For this reason, men's fitness may be less influenced by mode of spouse choice, while women face stronger tradeoffs between the potential genetic fitness benefits of independent mate choice and the fitness benefits derived from the socioeconomic support tied to arranged marriages. If the socioeconomic benefits of arranged marriage outweigh the benefits of independent mate choice, then women entering arranged marriages may be making an optimal choice that is better for their fitness and wellbeing.

The choice to have an arranged marriage, love marriage or elopement may also be heavily dependent on other factors like personality or socioeconomic status. For example, individuals whose personalities are more outgoing or independent may be more likely to choose their own spouses (e.g. Supplementary Information, quote A1), exert more control over fertility decisions, and also argue with in-laws. Additionally, individuals with low socioeconomic status may have less to lose by choosing their own spouse, and thus may be more likely to take that route. If this is the case and marriage types are not randomly distributed (which is likely), then the qualities of the individuals choosing particular types of marriages may be the reason for any fitness differentials, and this may confound the relationships between the genetic, social and financial benefits of different modes of spouse choice and fitness outcomes, as seen in other studies investigating to connections between marriage and fitness outcomes (e.g. Lawson & Gibson, 2018). Thus, further research should also investigate the characteristics of the individuals entering into different types of marriages.

### E. Limitations

While a mixture of convenience and snowball sampling allowed us to improve group dynamics and increase discussion, it could also limit the diversity of viewpoints. There were also differences in openness across groups. Unmarried individuals, especially women, were more reserved in sharing their opinions about marriage and relationships, possibly because dating and pre-marital relationships are not readily accepted in society. We attempted to circumvent this issue by asking questions about the community and age group generally, but there may still have been some hesitancy to speak openly. Recruitment may have also been more effective for certain socioeconomic or demographic groups. Attempts were made to recruit in various neighbourhoods and at varying times of day to mitigate this, but groups with different demographics may have offered alternative views.

Because many of the questions focused on what is typical in the community rather than personal experiences, we probably missed some variation. While many participants discussed their own experiences unprompted, it is likely that these personal anecdotes reinforced culturally acceptable opinions, while those that do not conform were probably less likely to be shared. We attempted to address this by asking specific questions about socially taboo topics, such as dowry and inter-caste marriages. Likewise, opinions or norms stated in these focus groups may describe ideal situations and not reflect actual behaviour in the community.

Because the ways in which these behaviours can affect reproductive and health outcomes are not always consciously understood, these groups do not offer a direct measure of how marriage type and fitness are connected. Quantitative measures of a wide range of variables would be required to draw conclusions about relationships bwteeen marriage type and fitness. These focus group discussions establish the ethnographic context of marriage in this community. From these data, we can make informed predictions about the potential for and avenues through which spouse choice may influence fitness.

## V. Conclusion

Previous studies attempting to examine the fitness effects of arranged and non-arranged marriage as proxies for limited and free mate choice, respectively, have largely returned null results. This may be due to the oversimplification of marriage type, the overestimation of parent–offspring conflict over mate qualities, the presence of confounding variables affecting both the likelihood of experiencing one type of marriage and reproductive outcomes, and/or the compensatory benefits that arranged marriages provide. From these focus group discussions it is clear that ‘Who chose your spouse?’ does not yield neatly dichotomous answers, and treating responses as such would obscure important variation in this community's spouse-choice dynamics. While parent–offspring disagreement over spouse qualities has been broadly detected in a variety of cultures around the world, these focus groups indicate less disagreement than expected and greater willingness between parents and offspring to compromise. The level of parent–offspring agreement appears higher for men than for women, indicating that women, who are also more likely to experience arranged marriages, may have more limited options in arranged marriages than do men. Thus, any study seeking to understand the fitness consequences of marriage types should first investigate the degree and the sources of parent–offspring disagreement in the community of interest. It is also clear from these discussions that arranged marriages may offer some compensatory benefits, such as increased social, financial and grandparental support that could plausibly affect fitness. Added familial financial support in arranged marriages, especially when it comes early in marriage, may lead to differences in the timing of reproduction. In this community the consequences of marrying against parents’ wishes appear harsher for women than for men, and thus different fitness effects may be seen depending on whether the bride's, groom's or both sets of parents disapprove of a marriage. Based on these results, it is clear that comparison of arranged and non-arranged marriages is not a clear proxy for limited and free mate choice, as defined by experimental animal studies. Studies seeking to assess the fitness consequences of marriage type will need to consider the potential benefits and costs of those marriage types and the demographic characteristics of individuals in those marriages in order to make culturally appropriate, context-specific predictions.

## Research transparency and reproducibility

Additional selected quotations and data summaries are available in the online Supplementary Information. The full transcripts of the focus group discussions used for this study are not yet publicly available because they constitute an excerpt of research in progress but are available from the corresponding author upon reasonable request.

## Supplementary material

For supplementary material accompanying this paper visit https://doi.org/10.1017/ehs.2023.3. click here to view supplementary material
