# Supplementary material for: Socioeconomic benefits and limited parent–offspring disagreement in arranged marriages in Nepal
Source: Evol Hum Sci. 2023 Feb 22;5:e7. doi: 10.1017/ehs.2023.3 (PMC10426041; doi:10.1017/ehs.2023.3)
Supplement: Supplementary file 1 [file S2513843X23000038sup001.docx]

**Supplementary Material**

Quotations excluded from the text

A: Is Spouse Choice a Dichotomous variable?

A1: “If they are the kind of daughters that listen to their family, then most of the time, they would do arranged marriage. But if they are *chanchal* [more extroverted] and have seen the city and city life, then those kinds of girls tend to do love marriage.” -Unmarried woman

A2: “But nowadays, in most of the cases, even if it is love marriage, the kids make sure that they introduce each other’s family and make them acquainted. And they will marry according to the traditions. What we can say is even though it is a love marriage everyone is happy since the permission has been asked to the concerned people in the family.” -Older woman

A3: “Today’s generation knows a lot. Both will check each other’s background regarding their lifestyle and what they want to do in their life [when they choose their own spouse]. They will see how much love they can give to each other and stuff like that.” -Older man

A4: “Once they [run away and] get married then slowly, they will form positive relationships with both of the families. It is going to take some time, but time heals everything. Eventually the son-in-law will get the love and respect that they are expected to get.” -Older man

B: Do parents and offspring agree on the ideal qualities of an in-law or spouse?

B1: “When they choose their life partner themselves, they don’t really look at the caste and religion but of course they will be checking for whether he/she can sustain the household and whether they are hard-working and can take responsibilities or not.” -Older man

B2: “If we want to marry someone from the lower caste family, then we have to convince our parents that they love us despite our caste. We should see if he is a hard-worker or not, and if he loves us or not, not what his caste is and how rich he is. Yes, this issue is still a lot in the society, but the society is slowly getting more progressive.” -Unmarried woman

B3: “We try to find beautiful girls for us but beauty will one day wither away, so this aspect normally comes last.” -Unmarried man

B4: “Beauty does not really matter as long as she is like average looking because I have seen some people who get married with an extremely beautiful girl, and one day the girl will just elope with another person who might be richer. So, it’s safer to marry an average looking girl.” -Unmarried man

B5: “When we look for a groom, we try to find a person who is gentle, hardworking, that does not have any affairs with other women, should have a job (especially a government job). If he possesses these qualities, then even if he is not that handsome it is fine because he can look after our daughter.” -Older man

B6: “We make sure that before we give our son or daughter for marriage, we ask the society around in which our sons and daughters are going to get married whether the family are good or not. If they have any bad aspects [we ask] what they are. We make sure that we understand the family clearly before giving our children to them.” -Older woman

B7: “If you do love marriage at a very early age, then it might be unsuccessful because you have not seen the whole world yet. And when you get more mature, then you have feelings that you want to date other people as well, and this could be the reason for divorce in love marriage if you do it early.” -Recently married man

B8: “In my time, I got married at 16, then I had a kid at 18, and now he is already 25 and I am 40 years old. I did not want to get married at that age, but as you know, we had to do what our parents and society told us to do. If I was young again, I would marry at 25 or 30 years old.” -Older woman

C: Do couples in arranged marriages receive compensatory benefits?

C1: “Here in Dhading district, I have found that even though you don’t have to give dowry, there are still some families that keep on hoping for something in each and every festival, rituals and ceremonies from the bride’s family. It is not clearly visible, but it happens in a lot of families.” -Older woman

C2: “I think dowry is increasing more nowadays. Lots of families in my society have not done their daughters' marriage because they are weak and can't give dowry.” -Recently married woman

C3: “In previous times, they used to give dowry because that was the trend in society here. That trend is still there, but nowadays people give because they want to give something to their daughter and son-in-law. It has drastically reduced compared to the previous time.” -Recently married man

C4: “I did not give any dowry in my marriage. Even at my daughter’s wedding, I will not be giving any dowry. I don’t need other people’s property [literal translation: to me other people’s property burns my body].” -Recently married woman

C5: “For me, there is no importance of dowry at all. With the dowries all you can do is decorate a room. It does not give you happiness or feed you. It does not matter how much we get the dowry but if we do not get a nice daughter in law with good behavior then all those dowries mean nothing. We need daughter in law with good behavior so that they can raise and support the family.” -Older woman

C6: “Regarding wealth distribution [inheritance] towards children from the parents, there is not any discrimination because government has made sure of it in the constitution.” -Unmarried man

Table S1: All desirable qualities discussed in each demographic. Abbreviations are as follows: IL= In-law, DIL= Daughter-in-law, SIL= Son-in-law, PR= Permanent resident

| Trait | Unmarried Women | Recently Married Women | Older women | Unmarried men | Recently married men | Older men |
| --- | --- | --- | --- | --- | --- | --- |
| Attractiveness | Mentioned as a desirable trait in spouse | Want DIL who is beautiful & humble  Matchmaker uses “handsome” as one feature to advertise an available spouse | SIL should be handsome & economically successful  DIL should be beautiful (among other qualities) | Should see whether a girl is beautiful or not before marriage  Beauty not the priority because it fades with age  Average looking is best because you don’t want girl to elope with someone richer  Look at the heart and not the face |  | Facial beauty not enough to choose a quality DIL  A SIL doesn’t need to be handsome if he can look after their daughter  Attractive mentioned with caste & horoscope as a desirable quality |
| Age gap |  | Big age gap (here, a groom 20 years older) will not produce a successful marriage  Should find a spouse similar in age |  | Wants a girl 2-3 years younger |  | Should be 3-4 year gap between bride and groom |

Table S1, continued

| Trait | Unmarried Women | Recently Married Women | Older women | Unmarried men | Recently married men | Older men |
| --- | --- | --- | --- | --- | --- | --- |
| Age of marriage | 24-25 years, 25 years  Want to pursue education first, marry after college and when they can make money on their own  20-25 years  Only want to marry once independent and have a career, marrying young might lead to a bad life because you’re not mature enough to make good decisions.  Wants to marry at 40 years old | 22-23 years old  When you marry after 20 years old, then the wife and babies will be healthier. Marrying as a teen isn’t good.  Mother’s and son’s health is affected if you marry too early  Only after 20 years old  20-25 years  People marry at earlier ages in love marriage  21-22 years old  I married at 15 but 25-26 is better | Kids like to go abroad for 3-4 years and then marry. Can’t marry until at least 20 years old, so 20-26 years is the window.  Marrying young caused difficulties  Unless kid asks they don’t initiate conversations about marriage  No one should marry in their teens; they should have a job and be independent  25, 22-25, 20-25 years old | When you’re 17 and have a beard/ mustache you’re getting mature enough to marry  In educated families, they want sons to finish school first. In villages/poor families men marry younger  If you don’t want to marry yet but family pressures you, some might commit suicide.  90% of men do what they want. 10% do what their parents want.  Marry after education (after +2/undergrad) or when you have a job. | When you see your friends get married then it’s your time.  If the family has problems they might pressure marriage earlier.  Getting a love marriage young won’t be successful.  You marry after all your elder brothers and sisters are married. For this person he was in his mid-30s. | After 22 years old because education is complete and people get jobs. There should be 3-4 years between bride/groom.  Whenever parents say it’s time to marry then the kids will marry.  Gov’t doesn’t allow marriage until 21 years.  After 16-17 years old they’ll start feeling attraction for other people, and this indicates they’re proceeding to the age of getting married. |

Table S1, continued

| Trait | Unmarried Women | Recently Married Women | Older women | Unmarried men | Recently married men | Older men |
| --- | --- | --- | --- | --- | --- | --- |
| Age of Marriage, continued | After 30 years because she wants to focus on career first and become independent  23 years, because religion requires marrying younger  Parents will start looking when they turn 20 or 21, but they don’t want to marry until 30 years old | Marrying after 20-21 years is not a good idea  22 and above, regret marrying young  In love marriage they fall in love young and get married hastily which isn’t successful  If they marry before 15 they’ll have all kids of physical problems.  When young kids are married they don’t have plans for the future and that causes conflict. | Parents want to marry their kids ASAP so they can have grandkids; it’s a child’s duty to marry before their parents die  22-26 years, 25-30 years old  Whatever age the kid wants to marry is fine, the family has to have consensus  Love marriage only works if they’re both mature enough (not super young) | When parents are getting old, you get married because they pressure you. Some elope in 9-10^th^ grade.  If you fall in love as a teen, then you do love marriage. Many people find love in undergrad. A lot of people regret marrying as teens.  Most people don’t marry unless they have jobs because otherwise their life will be hard.  18-19 year olds elope because they’re “young with hot blood” | When you see kids going out, dressing up, looking good then it might be time for them to marry. For girls, when they want to wear makeup and dress up then it’s time. | For sons, parents won’t pressure them to marry but for daughters you will get pressure.  Girls don’t get married at older ages because when you’re old you don’t tend to get the men you want.  Some girls want to marry after they do their masters and get a job.  16-17 year olds will do love marriages but if they’re more mature they’ll take their time and get permission from their parents first. |

Table S1, continued

| Trait | Unmarried Women | Recently Married Women | Older women | Unmarried men | Recently married men | Older men |
| --- | --- | --- | --- | --- | --- | --- |
| “Bad habits” (drinking, smoking, gambling) | Being a drunkard leads to worse marriages  Parents will look for someone who doesn’t drink/ smoke (x2)  They can smoke, but no drinking  Drinking and smoking doesn’t make someone bad unless it affects their personal life  Fighting over drinking/smoking can make a family bad  Some people chosen by parents can have bad habits, so choosing by yourself is the better option. | Being a drunkard is not good, even if the family is rich or has property.  Marrying a rich man who gambles, drinks, and cheats could result in wife’s suicide, separating, or divorce.  A SIL should not drink  Parents will seek a groom who doesn’t drink  Don’t marry a man who is an alcoholic | Will make sure a love match doesn’t have bad habits before approving the marriage.  SIL should not be an alcoholic.  A bride will regret an arranged marriage if she later finds out the groom is an alcoholic. | To get a girl to agree to marriage, families will advertise their sons as not drinking or using drugs.  A girl should not drink alcohol.  Not being an alcoholic would make a more successful marriage.  A girl’s family will make sure a potential groom does not take drugs or have any other bad habits. | (Not Discussed) | Will check to make sure potential in-laws don’t do drugs; even if the caste and education are fine, drug use is also important to check. |

Table S1, continued

| Trait | Unmarried Women | Recently Married Women | Older women | Unmarried men | Recently married men | Older men |
| --- | --- | --- | --- | --- | --- | --- |
| Caste | Lower-caste person will face discrimination, but will still try to convince parents if they are in love.  Nepali parents want same-caste in-law  Mentioned as first thing parents want  2 of 3 said they want same caste/religion; one person said they don’t care about society so they want an intercaste marriage  Will marry within caste for the happiness of their family | Bride/groom’s caste and religion should match | Castes should match  SIL can be of any caste  Won’t accept bride/groom of lower caste  Society will not approve of lower caste, but if a couple can’t live without each other they have to accept it. | Wife of lower caste will face discrimination in the household.  Parents will look at a girl’s caste.  Will look at potential bride’s caste first.  Want a bride with similar caste and same religion.  Parents will first look at caste, culture, religion. |  | First step is to make sure DIL is from same caste.  SIL should be of the same caste.  Children choosing for themselves don’t really look at caste. (mentioned twice)  Looking for qualities and skills but also caste.  Caste is starting to get outdated, but horoscope still matters. |

Table S1, continued

| Trait | Unmarried Women | Recently Married Women | Older women | Unmarried men | Recently married men | Older men |
| --- | --- | --- | --- | --- | --- | --- |
| Compatibility and understanding between the couple | Couples should keep each other happy, respect each other, and understand each other.  Should not get married right away; should take time to understand each other before marriage.  Parents want a SIL who loves them and doesn’t give them difficulties in life.  Potential spouse should be understanding.  Husband should be loving, caring, shouldn’t doubt us. | (Not discussed) | Make sure a potential love match is “perfect for my kids or not” by asking lots of questions.  Will look at whether couple can live happily together or not when looking for a S/DIL. | Want similar life goals/interests with wife.  They should know each other’s behavior before marriage so they know if they can stay together forever.  Understand each others’ stress and feelings and how to resolve issues.  Mutual respect & understanding makes marriage successful.  If in a relationship before marriage, will know good/bad aspects and whether they can support the other’s interests/ hobbies/career. | (Not discussed) | When choosing own spouse, they make sure lifestyles and life goals match up.  DIL should be understanding.  SIL should be understanding. |

Table S1, continued

| Trait | Unmarried Women | Recently Married Women | Older women | Unmarried men | Recently married men | Older men |
| --- | --- | --- | --- | --- | --- | --- |
| Compatibility and understanding between the couple, continued | Right spouse should be loving, understand her family situation  Want a spouse who will love them, treat them right, take care of them. |  |  | Make sure long-term plans are aligned.  Needs to be understanding in any circumstances they face for marriage to succeed.  No deep hatred between each other. |  |  |
| Economic success and/or high income | May use wealth to convince parents to allow marriage with lower-caste man.  Listed as desirable quality for both self and parents. | People with big age gap that marry for wealth are not successful.  Instead of wealth, husband should be humble and hardworking. | In-law’s family should be on same economic level.  Should make sure they get a good job after marriage. Should help an in-law get a job if they can’t.  SIL should sustain the family and give daughter a better future. | Parents will use girl’s family’s wealth to get son to marry her.  Girl’s family should be financially stable.  Some boys look at girl’s economic status instead of beauty. |  | SIL should have similar economic status and be able to sustain his family independently.  SIL should have a job (preferably gov’t job), and that’s more important than looks. |

Table S1, continued

| Trait | Unmarried Women | Recently Married Women | Older women | Unmarried men | Recently married men | Older men |
| --- | --- | --- | --- | --- | --- | --- |
| Economic success and/or high income, continued | Spouse should be able to provide for me/take care of me (both parents and offspring care about that)  Economic success is a necessity. Doesn’t have to be rich but should be able to pay for a basic house/apt/bills and won’t have to struggle for livelihood. | An in-law should start working after marriage so they can be financially independent.  Parents will look for someone who can economically sustain the household. | A SIL can be poor as long as he is educated and can get a good job. | Will look at whether they’re rich (after caste/horoscope and hard-working).  Girl should be something in her life or profession.  Should not choose someone based on being rich.  Both members of the couple should excel in career. |  | SIL should be hard working and have a job. |

Table S1, continued

| Trait | Unmarried Women | Recently Married Women | Older women | Unmarried men | Recently married men | Older men |
| --- | --- | --- | --- | --- | --- | --- |
| Education | (Not discussed) | Will look at potential bride/groom’s education.  IL should be educated  Parents will try to find a match who is same education level as their kid.  Lami uses educated as an advertisement for potential groom.  Man should be educated | Looking for a DIL with bachelor’s degree.  SIL can be poor but should be educated so he can get a good job.  Have to tell groom’s family if daughter will pursue education after marriage before moving forward with marriage plans.  DIL should be educated and civilized. | Couple’s education should match (either both uneducated or both educated) | (Not discussed) | Couple should have same education level, otherwise they’ll have misunderstandings between them and will have trouble running the household.  Education and skills important.  DIL should be educated, but also possess other qualities to keep household happy because DIL will take care of them in old age. |
| Faithful to partner | “he should never leave me and should not doubt me” | Marrying a playboy will result in bad marriage. |  |  |  | Look for a SIL that does not have affairs with other women. |

Table S1, continued

| Trait | Unmarried Women | Recently Married Women | Older women | Unmarried men | Recently married men | Older men |
| --- | --- | --- | --- | --- | --- | --- |
| Family Reputation, Affluence, Status | Parents will look for spouse from good family (listed as first quality x3)  Parents want spouse from a family who is respected  Families that fight are bad; smoking and drinking can lead to fighting  Lami will advertise a household as rich or high-status to make a man seem like a good match.  Parents want a spouse from a family with property, income | Parents will look at groom’s property and family history, and it doesn’t matter if the guy is good.  Look at IL’s family status in society.  Parents check groom’s property, family, and family history.  Family’s wealth can’t overcome other bad habits of a groom.  Should not marry on wealth alone.  Will check family status in society before marriage. | Before marriage, they will ask around about whether the family is good or not, and what their bad aspects are.  IL should be from civilized family.  Will make their kids understand that the spouse’s family is a “well-being family” if they are hesitant to marry.  Other family should be on same economic/social level.  Check potential IL’s family house and property. | Will check to see if a bride has “some standard in society or not”  Will persuade a son to marry a girl by saying she’s from a wealthy/high-class family.  Look at woman’s family status in society and financial stability  Will look at whether potential wife’s family is affluent or not.  Bride’s family should be affluent in society, even if they’re the right caste and wealthy. |  | Do a full background check of the family before initiating conversation about marriage.  Start conversation with family only if they have a good background.  Will check economic and social background of a potential bride or groom.  Will want daughter to marry guy from a rich family. |

Table S1, continued

| Trait | Unmarried Women | Recently Married Women | Older women | Unmarried men | Recently married men | Older men |
| --- | --- | --- | --- | --- | --- | --- |
| Freedom/ Autonomy | Groom should let us hang out with friends freely. | (Not discussed) | (Not discussed) | (Not discussed) | (Not discussed) | (Not discussed) |
| Gossip | (Not discussed) | An IL should not go around the village and start talking about their issues at home. | (Not discussed) | (Not discussed) | (Not discussed) | (Not discussed) |
| Happiness | Should find someone who can keep their partner happy (respect and understanding) | (Not discussed) | All we want is for children to be happy.  Will be happy no matter what type of marriage kids have. | (Not discussed) | Girl’s parents expect SIL to make daughter happy (love, take care of her) | (Not discussed) |

Table S1, continued

| Trait | Unmarried Women | Recently Married Women | Older women | Unmarried men | Recently married men | Older men |
| --- | --- | --- | --- | --- | --- | --- |
| Hard-working | Lami uses hard-working to advertise potential groom.  Hard-working is better than showing off.  Marry a man who isn’t hard-working will have unsuccessful marriage.  Will say groom is hard-working if trying to convince parents to approve lower-caste groom. | Want a hard-working and humble man.  Hard-working more important than wealth because hard work makes money.  SIL should be honest and hard-working.  IL should be hard-working, educated, and honest.  Parents will look for SIL who is hard-working  Groom should be hard-working | SIL should be hard-working and good in society.  DIL should be hard-working, respects ILs, patient, and happy in all conditions. | Families may exaggerate son’s hard-working to a bride and her family.  Bride should be hard-working (4 times) | DIL has to handle day-to-day household work nicely.  DIL should run household, even after ILs die. | DIL should carry out daily household duties.  When kids choose own spouse they’ll see if the spouse can sustain the household and whether they’re hard-working and can take responsibilities.  SIL should be hard-working so he can care for daughter.  DIL should know her responsibilities and is more welcomed if she has a job.  SIL should be hard-working and have a job. |

Table S1, continued

| Trait | Unmarried Women | Recently Married Women | Older women | Unmarried men | Recently married men | Older men |
| --- | --- | --- | --- | --- | --- | --- |
| Healthy | (Not discussed) | (Not discussed) | (Not discussed) | Make sure a bride is healthy and doesn’t have any potential diseases or long-term health issues. (x2) | (Not discussed) | (Not discussed) |
| Honesty | A good SIL should be honest and lovable  Mentioned as a desirable quality in a spouse | SIL should be honest and hard-working  IL should be educated and honest | (Not discussed) | Family will tell bride’s family son is honest & hard-working.  Bride should be honest, respect & love rest of family  Couple should be honest with each other.  Bride should be honest & hard-working. | (Not discussed) | DIL should serve and respect parents and have an honest personality. |
| Horoscope | (Not discussed) | (Not discussed) | (Not discussed) | Parents will look at astrological and birth chart of potential bride.  First, look at caste, astrology, culture. (x2) | (Not discussed) | Look at IL’s caste and horoscope x2  Caste is getting outdated, but horoscope/ astrology still matter. |

Table S1, continued

| Trait | Unmarried Women | Recently Married Women | Older women | Unmarried men | Recently married men | Older men |
| --- | --- | --- | --- | --- | --- | --- |
| Humble | Like hard-working guys better than ones that show off. | Want hard-working & humble man  A humble & good man can sustain his family, but a drunkard cannot.  Hard-working and humble more important than wealth.  Want DIL who is beautiful and humble. | (Not discussed) | (Not discussed) | (Not discussed) | (Not discussed) |
| Immigration Status | (Not discussed) | (Not discussed) | (Not discussed) | If a girl has a green card or PR status, it’s a good way to get a guy to marry her.  Family will pressure marriage if the boy/girl has a green card. | (Not discussed) | If SIL is from Australia and has PR status then parents will want daughter to marry him no matter what because it’ll increase her standard of living. |

Table S1, continued

| Trait | Unmarried Women | Recently Married Women | Older women | Unmarried men | Recently married men | Older men |
| --- | --- | --- | --- | --- | --- | --- |
| Intelligence | (Not discussed) | Should make daughter educated and smart—that is her dowry. | (Not discussed) | Bride should be intellectual, moral, and do household chores. | (Not discussed) | SIL should be smart and understanding |
| Love/Loving/ Lovable | Our parents want a SIL who loves us and doesn’t give us difficulties  Parents will make sure guy loves her before approving a marriage.  Groom should love her and be understanding.  Groom should be loving, caring, & shouldn’t doubt us.  Couple should show some amount of love  Groom should love us and take care of us | If they love each other everything will be fine.  Any type of marriage will be fine if they like each other.  Parents will find someone who loves me.  A husband should love me. | SIL should love us (the parents), his wife, and respect us (parents)  DIL should love her husband and respect us (his parents).  If a DIL loves and respects us we will do the same. | If the heart falls in love with another soul, then there will be a marriage (more important than the qualities each of them have). | SIL should love daughter and make her happy. | Young people today will see how much love they can give each other before deciding to marry.  SIL should love our daughter. |

Table S1, continued

| Trait | Unmarried Women | Recently Married Women | Older women | Unmarried men | Recently married men | Older men |
| --- | --- | --- | --- | --- | --- | --- |
| Patience | (Not discussed) | (Not discussed) | DIL should be patient and happy in all conditions. | (Not discussed) | A potential spouse should have patience. | (Not discussed) |
| Respectful | Couple should respect each other. | (Not discussed) | SIL should love us, his wife, and respect us.  DIL should love her husband & respect us, not fight over small things, be patient, & be happy in any condition.  If a DIL loves and respects us, we will do the same.  D/SIL should love and respect us and consider both families equal when making decisions.  DIL should respect everyone, be *sanskari*, and not be greedy/ jealous | Wife should respect elders & love the young people in the family.  Wife should love & respect our parents (x2)  Will see whether potential wife has good traits, manners, and whether she respects people or not.  After getting married and coming to my house, a wife should love and respect my parents. | Parents expect that a DIL will have good relationships with other family members and society, even after they die. | DIL should serve and respect their parents, have an honest personality, have a pure heart, and love others.  DIL should have qualities that make the house happier (respect family members).  DIL should respect & love us because how else can we get old (and have them take care of us)? This is the most important quality.  SIL should know how to respect and interact with individuals in society. |

Table S1, continued

| Trait | Unmarried Women | Recently Married Women | Older women | Unmarried men | Recently married men | Older men |
| --- | --- | --- | --- | --- | --- | --- |
| *Sanskari* (Good manners, well-behaved, studious, doesn’t party, cooks/does chores, etc.) | (Not discussed) | (Not discussed) | DIL should be *sanskari*, respect everyone and not be greedy/  jealous. | (Not discussed) | (Not discussed) | (Not discussed) |
| Supportive | Parents will look for a groom who will stand by her in thick and thin | Husband should help me in my profession, and I should help him in his profession. | SIL should raise his family like a good husband. | Should support each other’s interests, hobbies, and careers. | Women should support their husband, make him go right way when he seems to be going the wrong way. | (Not discussed) |
| Well behaved | When introducing parents to a man they love, parents make sure guy has good behavior & loves the daughter.  Right marriage partner should have good behavior x2  Parents also want groom who has good behavior | (Not discussed) | SIL should be hard-working & good in society. Should not drink & raise family like a good husband.  DIL should have good behavior to raise and support family; dowry means nothing without good behavior. | Parents will look for a girl with good behavior who can carry out household chores.  Wife should love and respect ILs, be intellectual, moral, and do household chores. | Wife/DIL should behave nicely with other people in society. Should handle daily work nicely. Should positively influence husband’s behavior. | DIL should not be talkative, be understanding, not be notorious in society, know her responsibilities, and love the family. |

Note: Abbreviations in the above table include IL= In-law, DIL= Daughter-in-law, SIL= Son-in-law, PR= Permanent resident
